# Dose-Dependent Neuroprotective Effects of Bovine Lactoferrin Following Neonatal Hypoxia–Ischemia in the Immature Rat Brain

**DOI:** 10.3390/nu13113880

**Published:** 2021-10-29

**Authors:** Eduardo Sanches, Yohan van de Looij, Sadou Sow, Audrey Toulotte, Analina da Silva, Laura Modernell, Stéphane Sizonenko

**Affiliations:** 1 Division of Child Development and Growth, Department of Pediatrics, School of Medicine, University of Geneva, 1205 Geneva, Switzerland; ef.sanches@yahoo.com (E.S.); yohan.vandelooij@epfl.ch (Y.v.d.L.); sadou.sow@etu.unige.ch (S.S.); audrey.toulotte@unige.ch (A.T.); laura.malaguti@etu.unige.ch (L.M.); 2 Center for Biomedical Imaging (CIBM), Animal Imaging and Technology Section, Ecole Polytechnique Fédérale de Lausanne (EPFL), 1015 Lausanne, Switzerland; analina.dasilva@epfl.ch

**Keywords:** prematurity, lactoferrin, hypoxia–ischemia, neuroprotection

## Abstract

Injuries to the developing brain due to hypoxia–ischemia (HI) are common causes of neurological disabilities in preterm babies. HI, with oxygen deprivation to the brain or reduced cerebral blood perfusion due to birth asphyxia, often leads to severe brain damage and sequelae. Injury mechanisms include glutamate excitotoxicity, oxidative stress, blood–brain barrier dysfunction, and exacerbated inflammation. Nutritional intervention is emerging as a therapeutic alternative to prevent and rescue brain from HI injury. Lactoferrin (Lf) is an iron-binding protein present in saliva, tears, and breast milk, which has been shown to have antioxidant, anti-inflammatory and anti-apoptotic properties when administered to mothers as a dietary supplement during pregnancy and/or lactation in preclinical studies of developmental brain injuries. However, despite Lf’s promising neuroprotective effects, there is no established dose. Here, we tested three different doses of dietary maternal Lf supplementation using the postnatal day 3 HI model and evaluated the acute neurochemical damage profile using ^1^H Magnetic Resonance Spectroscopy (MRS) and long-term microstructure alterations using advanced diffusion imaging (DTI/NODDI) allied to protein expression and histological analysis. Pregnant Wistar rats were fed either control diet or bovine Lf supplemented chow at 0.1, 1, or 10 g/kg/body weight concentration from the last day of pregnancy (embryonic day 21–E21) to weaning. At postnatal day 3 (P3), pups from both sexes had their right common carotid artery permanently occluded and were exposed to 6% oxygen for 30 min. Sham rats had the incision but neither surgery nor hypoxia episode. At P4, MRS was performed on a 9.4 T scanner to obtain the neurochemical profile in the cortex. At P4 and P25, histological analysis and protein expression were assessed in the cortex and hippocampus. Brain volumes and ex vivo microstructural analysis using DTI/NODDI parameters were performed at P25. Acute metabolic disturbance induced in cortical tissue by HIP3 was reversed with all three doses of Lf. However, data obtained from MRS show that Lf neuroprotective effects were modulated by the dose. Through western blotting analysis, we observed that HI pups supplemented with Lf at 0.1 and 1 g/kg were able to counteract glutamatergic excitotoxicity and prevent metabolic failure. When 10 g/kg was administered, we observed reduced brain volumes, increased astrogliosis, and hypomyelination, pointing to detrimental effects of high Lf dose. In conclusion, Lf supplementation attenuates, in a dose-dependent manner, the acute and long-term cerebral injury caused by HI. Lf reached its optimal effects at a dose of 1 g/kg, which pinpoints the need to better understand effects of Lf, the pathways involved and possible harmful effects. These new data reinforce our knowledge regarding neuroprotection in developmental brain injury using Lf through lactation and provide new insights into lactoferrin’s neuroprotection capacities and limitation for immature brains.

## 1. Introduction

The high number of live preterm births, especially those born at very low birth weights (VLBW) (under 1500 g) younger than 32 gestational weeks, leads to subsequent central nervous system damage and developmental abnormalities [1]. The risk of neonatal brain injury due to peri- and/or post-natal hypoxia–ischemia (HI) is higher in preterm infants [1,2], and also predicts more severe prognosis compared to term babies [3,4]. Hypoxic–ischemic or inflammatory cerebral damage in premature infants, are the main causes of motor and cognitive disorders, cerebral palsy, autism, and attention-deficit hyperactivity disorder (ADHD), and are risk factors for psychiatric disorders in adulthood [5,6]. Currently, there is no specific treatment used to protect “preterm HI brains” [7]. Hypothermia is the unique therapeutic approach used, but is targeted to term hypoxic encephalopathy; however, nearly 50% of infants do not benefit [8,9] and there is no safe protocol for its use in preterm infants [10,11].

Experimental HI performed in rats on post-natal day 3 (HIP3) mimics HI occurring in human preterm infants born between 24 and 28 weeks, since animals at this stage of development present brain cellular development similar to humans at this age [12,13,14,15]. Tissue injury occurs preferentially in the white matter and in cortical areas, especially in the corpus callosum and parietal cortex of the ischemic hemisphere; however, other structures can be injured depending on the severity of hypoxia exposure [16,17,18]. Neuroinflammation with persistent astrogliosis [13,19], systemic upregulation of pro-inflammatory cytokines, microglial activation, blood–brain barrier leakage and oxidative stress are key features of the HI injury [20,21]. This results in cortical and subcortical damage, hypomyelination and altered cerebral microstructure leading to functional impairments [22,23].

There is robust preclinical evidence that supports the neuroprotective properties of nutritional interventions during pregnancy counteracting HI brain damage. The use of folic acid [24,25], docosahexaenoic acid [26,27] resveratrol [28], vitamin A [29], melatonin [30], as well as dietary interventions using pomegranate juice [31] or broccoli sprouts [32], and others, may represent a safe and inexpensive strategy for the prevention and treatment of neonatal HI damage. Lactoferrin (Lf) is an iron-binding glycoprotein found in breast milk [33,34,35]. After oral administration, Lf is regulated through binding sites on brain endothelial cells allowing the passage from blood to tissues including the brain [36]. Lf exhibits multiple biological effects, which are likely to decrease cerebral lesions: anti-inflammatory, anti-oxidant, anti-apoptotic (for review, see [37]). However, despite its beneficial effects, only a few research groups have been interested in its therapeutic potential for neuroprotection and support for brain development even in the presence of data showing that breast milk (in which Lf is highly abundant) reduces neurological outcomes induced by preterm birth, infections and necrotizing enterocolitis [38,39].

Regarding Lf actions on the central nervous system, previous studies have shown a neuroprotective effect of Lf given through lactation to the developing brain in rat models of perinatal hypoxia–ischemia and inflammation using LPS [40,41], as well as in IUGR models based on maternal caloric restriction [42] and prenatal corticoid exposure [43], in which anti-oxidative, anti-inflammatory, and neurotrophic properties were observed. These preclinical evidences using Lf as a neuroprotective nutrient in the developing brain is the foundation for transposition to future clinical studies. However, the optimal Lf dose as well as precise mechanisms of action remain to be better understood in order to go into clinical trials. Furthermore, it is assumed that a specific dose of Lf is required to achieve optimal neuroprotection following HI in very immature brains.

As such, the aim of this study was to establish the optimal neuroprotective dose of Lactoferrin in a model of preterm hypoxic–ischemic brain injury. Evaluating brain metabolism as well as structure and neurobiological substrates of neuroprotection at two different time points after HI injury, Lf supplementation has been evaluated for three different doses. Brain neurochemical profile and structure were assessed using magnetic resonance spectroscopy (MRS) in conjunction with protein expression in the acute phase after injury (24 h). Long-term brain damage was evaluated or determined using advanced diffusion magnetic resonance imaging techniques, diffusion tensor imaging (DTI) as well as neurite orientation dispersion and density index (NODDI), and histological assessments at P25.

## 2. Material and Methods

### 2.1. Animals

**Figure 1:**
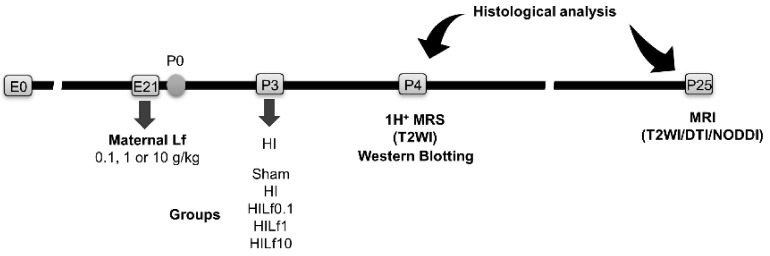
Experimental timeline of the study. Embryonic day (E). HI—hypoxia–ischemia. P, postnatal day. DTI, diffusion tensor imaging. Lf—lactoferrin. NODDI, neurite orientation dispersion and density index.

The Geneva State Animal Ethics Committee and the Swiss Federal Veterinary Service approved the project as #31629. Pregnant Wistar rat dams purchased from Charles River, France were fed with a diet supplemented with bovine lactoferrin (Lf) (holo-lactoferrin/LPS-free, Taradon laboratory, Tubize, Belgium) at an expected dose of 0.1, 1, or 10 g/kg (Provimi Kliba SA, Penthalaz, Switzerland) ad libitum from the last day of pregnancy during the whole period of lactation. Based on previous experiments [41], the average food consumption of a dam weighting 300 g during the first week of lactation is 35 g diet/day. Thus, the diet was enriched in Lf at 0.85% to ensure 0.3 g Lf per day (10-fold less for the Lf0.1 group and 10-fold more for Lf10 g/kg), corresponding to an expected dose of 1 g/kg/day). Control litters were fed with an isocaloric diet using casein as protein source. To reduce inter-variability within litters, all groups were processed in parallel for each set of experiments. Standard animal housing conditions (22 ± 2 °C, 12 h light/dark cycle) were applied with free access to food and water. On the first day of life (P1), rat pups were sexed to ensure as much as possible, 50% of each sex per group (Sham or HI) and limited to 10 pups per litter. Animals from the same litter were used for different assessments. Pup weight gain was measured daily from P3 to P6 and once a week until P25. The experimental schedule is presented in Figure 1. The dose–response curve for Lf supplementation was chosen to contrast the dosage used in previous papers from the group (1 g/kg of Lf) and have shown to produce no adverse effects in rats when administered to the mother during lactation [41,42,43]. The other two doses were then established according to a geometric sequence from the maximum with common ratio 1/10: 0.1 g/kg and 10 g/kg. Before the surgical procedure, animals were divided into five experimental groups according to maternal dietary supplementation; Control diet (*n* = 6), Lf0.1 g/kg (*n* = 7), LF1 g/kg (*n* = 6), and Lf10 g/kg (*n* = 7). Sham and HI had no Lf in the maternal diet; HILf0.1, HILf1, and HILf10 were supplemented with Lf in the respective dose.

### 2.2. Brain Hypoxia–Ischemia

HI was performed using the modified Vannucci HI at P3, as previously described, with slight modifications [23,40]. Briefly, 3-day-old male and female rat pups underwent unilateral right common carotid artery occlusion, and after a 30 min recovery period (in which animals were returned to their mothers), they were submitted to hypoxic atmosphere (6% O_2_ balanced in 94% N_2_) at 37 °C for 30 min. Sham-operated rats had neither common carotid artery ligation nor hypoxia exposure.

### 2.3. ^1^H-Magnetic Resonance Assessment

As previously described [23], magnetic resonance experiments were all performed on an actively shielded 9.4 T/31 cm magnet (Agilent/Varian/Magnex) equipped with 12-cm gradient coils (400 mT/m, 120 µs).

#### 2.3.1. ^1^H-Magnetic Resonance Spectroscopy (MRS)

Animals were scanned at P4 (i.e., 24 h post HI) with a homemade quadrature transmit-receive surface radiofrequency coil of 17-mm diameter. For MRS, groups were composed as follows: Sham: *n* = 8, HI: *n* = 10, HILf0.1 *n* = 10, HILf1 *n* = 10, and HILf10 *n* = 7.

Briefly, animals were placed under isoflurane anaesthesia (1.5–2.0%) in supine position in a homemade holder. Body temperature (37 ± 0.5 °C) and heart rate were monitored and regulated during MR acquisition [44]. A Fast Spin Echo T_2_W image was performed to position ^1^H-MRS voxel of interest. ^1^H-MRS spectra acquisition was performed on the right cortex (voxel of interest of 1.5 × 1.5 × 2.5 mm^3^) using an ultrashort echo time (TE/TR52.7/4000 ms) SPECIAL spectroscopy method. The spectra were acquired in 16 blocks of 16 averages after automatic FASTMAP shimming [45]. LC-model [46] was used to analyse the acquired spectra and quantify the following cortical metabolites: aspartate (Asp), alanine (Ala), ascorbate (Asc), creatine (Cr), phosphorylcholine (PCho), phosphocreatine (PCr), g-aminobutyric acid (GABA), glutamate (Glu), glutamine (Gln), glutathione (GSH), glycine (Gly), lactate (Lac), macromolecules (Mac), myo-inositol (Ins), N-acetylaspartate (NAA), N-acetylaspartylglutamate (NAAG), and phosphoethanolamine (PE).

#### 2.3.2. Diffusion Imaging (DTI-NODDI)

At P25 (Sham: *n* = 8, HI: *n* = 8, HILf0.1 *n* = 8, HILf1 *n* = 8, HILf10 *n* = 5), rats were sacrificed and brains were paraformaldehyde-fixed for subsequent ex-vivo MRI with a 2.5 mm diameter birdcage coil. Brains were kept at least six weeks in phosphate-buffered saline solution for stabilization before MRI. A multi b-value shell protocol was used based on a spin echo sequence with the following parameters: field-of-view of 21 × 16 mm^2^, 20 slices of 0.6 mm, 3 accumulations with TE/TR = 45/2000 ms. Moreover, 96 diffusion weighted images were acquired with 15 images with b = 0 and 81 images distributed as follows: 21, 30, and 30 directions with b-value equal to 1750, 3400, and 5100 s/mm^2^, respectively.

Acquired data were fitted with conventional diffusion tensor imaging (DTI) model using Diffusion Tensor Imaging ToolKit (DTI-TK) and with the neurite orientation dispersion and density imaging (NODDI) model using the NODDI toolbox [47]. For NODDI, a supplementary compartment (intra-restricted volume fraction, irfrac) was added corresponding to stationary water (i.e., without diffusion) following brain fixation. Regions of interest (ROI) were manually delineated in the corpus callosum (CC), external capsule (EC), Motor Cortex, Somatosensory Cortex, Internal Capsule (IC) and Caudate-Putamen (CP) on colour maps using homemade MATLAB scripts. DTI derived parameters were assessed based on templates 13–14 (for anterior region—data shown in Supplementary Figure S1) and 35–36 (for posterior region) obtained in the brain map atlas (http://larrywswanson.com/?page_id=164, last accessed on 27 October 2021). DTI derived parameters (diffusivities: mean: MD, axial: AD and radial: RD as well as fractional anisotropy (FA)) and NODDI derived parameters (intra-neurite volume fraction (fin), isotropic volume fraction (fiso) and orientation dispersion index (ODI)) were averaged for all these ROIs. Results are presented as mean ± SD. A mask was performed on MD images to obtain total brain volumes.

### 2.4. Immunoblotting

Pups were anesthetised with pentobarbital (100 μg/kg) and euthanized by decapitation at P4 and P25 (*n* = 4–8 animals/group). Right cortices and hippocampi were quickly removed, dissected out on ice, frozen instantly in liquid nitrogen, and stored at −80 °C until further analysis. Samples were thawed in RIPA buffer (Cell Signaling, 9806S) homogenised by sonication and protein concentration was assessed using the Bradford assay [42]. Proteins (25 μg) were separated using SDSPAGE, transferred to a nitrocellulose membrane, and analysed by fluorescent immunoblotting using the iBright FL1500^TM^ Imaging system to visualize protein bands. All antibodies were diluted in blocking buffer containing 0.1% casein (Sigma-Aldrich, C8654, Buchs, Switzerland) and diluted at per the concentrations suggested in the data sheet by the manufacturer (see Table 1 for the antibodies used). After overnight incubation with primary antibodies, rinsing with PBS Tween20 was performed. Membranes were then incubated with secondary antibodies: goat anti-mouse IgG (H + L) conjugated with DyLight^TM^ 680 (#5470, Cell Signaling Technology, Allschwil, Switzerland), goat anti-rabbit IgG conjugated with IRDye^TM^ 800 (#926-32210, LI-COR^TM^, Bad Homburg, Germany). The densitometry was assessed by normalizing the optical density of each sample and results are expressed as the average of values obtained for the Sham group (set 1) using actin or ßIII tubulin expression as reference, using Image StudioLite™ version 5.2.

### 2.5. Immunofluorescence

Animals were perfused intracardially with PBS and 4% paraformaldehyde (PFA), brains were removed from the skull and post fixed overnight, then dehydrated in 30% sucrose and frozen in isopentane at −20 °C for posterior slicing using a cryostat (Thermo Fisher—*NX50*, Basel, Switzerland). In this study, each fifth serial brain coronal 30-μm slice from 3 to 5 animals per group were assessed. P4 brains were assessed from −0.40 to −1.20 mm, from Bregma levels according to brain coordinates from the developing brain atlas [48]. Tissue collected at P25 was sectioned following ex-vivo MRI between the −2.3 and −3.3 mm corresponding areas to the Paxinos atlas (*n* = 3 animals/group) [49]. Glial fibrillary acidic protein (GFAP) (astrocytic marker; 1:500, 6171—Sigma-Aldrich, Buchs, Switzerland), NeuN (neuronal marker; 1:300, MAB377—Chemicon International, Inc., California, US), MBP (myelin; 1:300, ab62631—Abcam, Geneva, Switzerland), and Iba-1 (macrophage—microglial marker; 1:250, ab507, Abcam, Geneva, Switzerland) were quantified. At P4 (*n* = 3 animals/group); samples were double-labelled using NeuN or GFAP with cleaved caspase-3 (apoptotic cell death marker; 1:300, 9661—Cell Signalling, Allschwil, Switzerland) in order to identify cell-specific apoptotic death. At P4, astrocytes (GFAP) were double-labelled with cleaved caspase 3 in order to identify CC astrocytes apoptotic cell death. Co-localization of ODI was performed using Coloc2 plug-in and results are expressed as the mean of Pearson’s correlation R-value. Briefly, all slides were washed in PBS and blocked for 45 min with 3% bovine serum albumin (BSA; Sigma-Aldrich, Allschwil, Switzerland) in PBS, with 0.3% Triton X-100 at room temperature. Then, the slides were incubated overnight with primary antibody at 4 °C in PBS, 0.3% Triton X-100, and 3% BSA. On the following day, slides were washed in PBS and incubated in the dark with secondary fluorescent antibodies for 2 h at room temperature, mounted and cover-slipped with anti-fading mounting acidic mounting media containing DAPI (FluorSave™—Sigma, Allschwil, Switzerland). IgG rabbit or mouse secondary antibodies, Alexa Fluor 488 or 555, were used. The primary antibody was omitted on some Sham animal slides for immunostaining negative controls. Images were acquired using a Nikon Eclipse T2000 microscope (Japan) coupled to a DS-Qi2 camera using a 10× objective. Motor and somatosensory cortices, CA1, and dentate gyrus (DG) were assessed using the same region of interest (200 μm^2^) for each slice, and the optical fluorescence intensity (ODI) was quantified using NIH-ImageJ software (version 1.8.0) by an experimenter blinded to the groups. Corpus callosum thickness was measured at two distinct levels in the dorsal hippocampal in the same slides used for MBP immunofluorescence analysis.

### 2.6. Statistics

Statistical analysis was performed using SPSS™ software version 21. The number of animals used is presented for each technique assessed and in the corresponding graph. It is important to note that animals were obtained from at least 3 different litters and were chosen randomly for either timepoint or technique. Repeated measures ANOVA was used to assess body weight of animals during development. Normally distributed data were analysed by one-way ANOVA followed by Duncan’s post hoc. Kruskal–Wallis test followed by the Mann–Whitney test was performed in case of non-normal distribution of data. Data are presented as mean ± SEM except for DTI and NODDI derived parameters, which are expressed as mean ± SD. Significance level was considered when *p* < 0.05. *P*-values between 0.05 and 0.1 were mentioned since they may indicate trends that are biologically relevant [11].

## 3. Results

### 3.1. HI Causes a Decrease in Body Weight Not Reversed by Lf

Body weight was measured daily from P3 to P6 and at P11, P18, and P25 (before sacrifice) (Table 2). Repeated measures ANOVA using time and group as variables showed an interaction between factors (F(4,99) = 3.89 *p* = 0.006) in which HI animals, despite Lf treatment, had smaller body weight compared to the Sham group. The test between subjects (F(4,99) = 2.746 *p* = 0.03) revealed that HILf1 g did not differ from Sham animals over time evidencing an optimal effect of Lf at 1 g/kg for this variable.

### 3.2. Lf Reduces Brain Metabolic Deficits in the Cerebral Cortex Following HI

**Figure 2:**
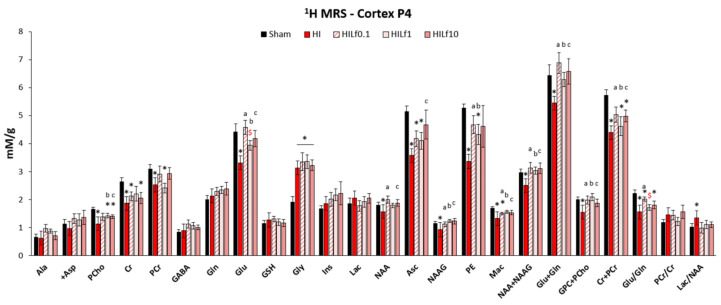
Neurochemical profiles presented as mean ± SEM. All concentrations are expressed in mM/g. Differences between groups (Sham, HI, HILf0.1, HILf1, and HILf10) 24 h post-HI (*p* < 0.05, * HI vs. SH, ^$^ difference between HILf groups. ^a b c^ HILf0.1, 1 or 10 vs. HI, respectively. Differences were determined by one-way ANOVA and considered significant when *p* < 0.05. Asc, ascorbate; Ala, alanine; +Asp, aspartate; Cr, creatine; GABA, gamma-aminobutyric acid; Gln, glutamine; Glu, glutamate; GSH, glutathione; Gly, glycine; Ins, myo-inositol; Lac, lactate; Mac, macromolecules; NAAG, N-acetylaspartylglutamate; NAA, N-acetylaspartate; PCho, phosphocholine; PCr, phosphocreatine; PE, phosphoethanolamine.

Modifications induced by HI in the neurometabolic profile were studied in the cerebral cortex in order to describe the acute neuroprotective effects of Lf 24 h after brain injury. Metabolite concentrations are shown in Figure 2. FASTMAP shimming (first-order and second-order correction of the magnetic field homogeneity) enabled to obtain very good-quality spectra in a volume of 12 µL in the parietal cortex. The average signal-to-noise ratio calculated on all acquired spectra was 12.3 ± 2.2. HI induced a significant decrease in the concentrations of PCho, Cr PCr, Glu, NAA, Asc, NAAG, PE, Mac, Tau, NAA + NAAG, Glu + Gln, GPC + PCho, Cr + PCr, and the Glu/Gln ratio, similar to previously reported data [23,40]. Lf supplementation through lactation was able to partially reverse most of the alterations induced by HI insult. Importantly, although all three doses reduced metabolite alterations induced by HI, the effects in Cr and PCr, as well as in Glu + Gln and Glu/Gln were clearly dependent of the Lf dosage, which may infer that the mechanisms of Lf neuroprotection could be distinct depending on the dose used [50]. HILf0.1 had the best performance, decreasing alterations induced by HI in the Cr + PCr and Glu/Gln ratio. Although not significant, the increase in Gln and Asc observed in HILf10 animals could suggest an answer of the antioxidant system counteracting the effects of HI due to an increase in oxidative species. Importantly, all three doses restored Lac/NAA levels (a ratio used as an early biomarker of HI injury, since it measures both mitochondrial impairment and neuronal integrity) to levels similar to the Sham group, evidencing neuroprotective effects of maternal Lf supplementation.

### 3.3. Molecular Substrates of Lactoferrin Neuroprotection

**Figure 3:**
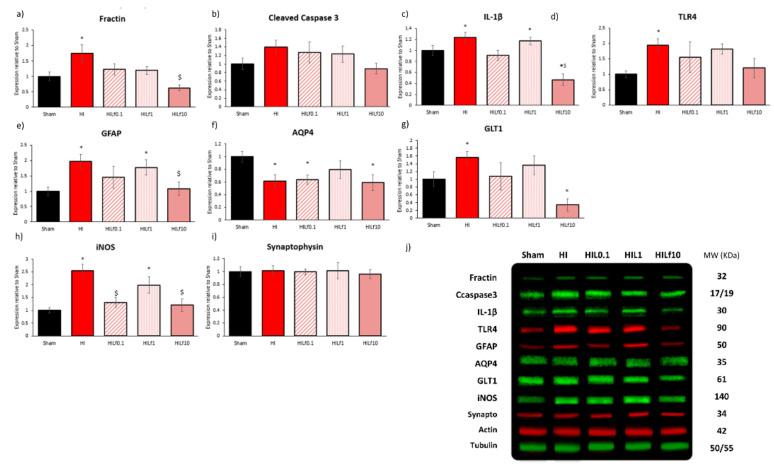
Effects of Lf and HI on protein expression in the right cortex at P4 (24 h) after injury. Dams were fed control diet (Sham and HI), Lf 0.1 (HILf0.1), 1 (HILf1), or 10 (HILf10) g/kg lactoferrin. All proteins were quantified by densitometry, and then normalised to actin or tubulin and expressed as a value (ODI) relative to the Sham group for: fractin (**a**), cleaved caspase 3 (**b**), IL-1β (**c**), TLR4 (**d**), GFAP (**e**), AQP4 (**f**), GLT1 (**g**), iNOS (**h**), and synaptophysin (**i**). Representative bands of proteins with the respective molecular weight (mw) in KDa (**j**). All values are presented as the mean ± SEM, *n* = 4–6 animals per group. * HI vs. SH, ^$^ difference between HILf groups. Differences were determined by one-way ANOVA and considered significant when *p* < 0.05.

Lf-diet-mediated effects on brain neurochemical profile, decreasing HI injury, with a distinct pattern of neuroprotection according to the dose of lactoferrin used. Thus, proteins related to cell death (fractin and cleaved caspase 3), neuroinflammation (IL-1β, TLR4), oxidative stress (iNOS), astroglial reactivity and glutamatergic reuptake (GFAP, GLT1 and AQP4), and synaptic transmission (synaptophysin) were assessed in the ipsilateral cortex (Figure 3) and hippocampus (Figure S1) 24 h after injury. In the right cortex, protein expression of inflammatory filament alteration marker Fractin ((F4,31) = 5.22 *p* = 0.003), and markers of inflammation IL-1β ((F4,23) = 8.80 *p* < 0.001) and TLR4 ((F4,23) = 2.88 *p* = 0.05) were increased in HI group and reversed in treated animals (Figure 3a,c,d) mainly with Lf10 g/kg. No differences in cleaved caspase 3 expression were observed (Figure 3b). Astroglial reactivity marker GFAP was increased in HI groups and decreased with the highest dosage of Lf used ((F4,31) = 5.87 *p* = 0.02); the same pattern was observed for GLT-1 ((F4,22) = 3.06 *p* = 0.02) (Figure 3e,g). AQP4 was decreased in HI groups, except HILf1 g/kg ((F4,23) = 3.31 *p* = 0.03) (Figure 3f). iNOS expression was increased in HI and HILf1 g/kg and reversed with 0.1 and 10 g/kg of Lf (F(4,26) = 4.41 *p* = 0.009) (Figure 3h). No difference was observed in Synaptophysin 24 h after HI injury (Figure 3i). As shown in Figure S1a–k, hippocampus was less injured due to HIP3. However, in this area HI animals presented an increase in cleaved caspase 3 (F(4,31) = 3.08 *p* = 0.03) reversed by all three doses of Lf (Figure S1b). Regarding the effects of Lf, HILf10 caused a decrease in IL-1β ((F4,23) = 4.50 *p* = 0.01) (Figure S1c) and trends to increase GFAP and iNOS (Figure S1e,h). Allied to this, the HILf10 group shows an increase in Synaptophysin ((F4,23) = 3.63 *p* = 0.02) (Figure S1i). The HILf0.1 group had increased expression of AQP4 ((F4,23) = 7.98 *p* = 0.001) and DCX ((F4,23) = 3.26 *p* = 0.03) compared to the other groups (Figure S1f,k).

### 3.4. Long-Term Brain Macro and Microstructure Evaluation

**Figure 4:**
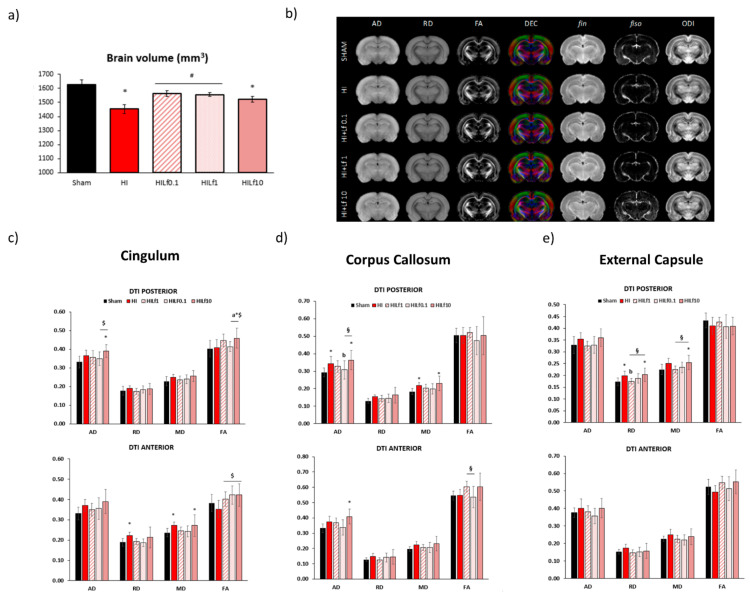
MRI data at P25. Left upper panel (**a**)—brain volumes (mm^3^) (*n* = 5–8 animals/group). Right upper panel (**b**)—Diffusivity (mean, MD; axial, AD; radial, RD), fractional anisotropy (FA) and direction encoded colour (DEC) maps, intra-neurite volume fraction (fin), cerebrospinal volume fraction (fiso), and orientation dispersion index (ODI) maps. Maps correspond to the averaged maps over each group. Lower panels: histogram of diffusivities (mean, MD; axial, AD; radial, RD; ×10^−4^ mm^2^·s^−1^) and fractional anisotropy (FA) in the cingulate gyrus (left panels—**c**), corpus callosum (central panels—**d**) and external capsule (right panels—**e**). Results are mean ± SD. * HI vs. SH, ^$^ difference between HILf groups. ^a b^ HILF0.1, or 1 vs. HI, respectively. Differences were determined by one-way ANOVA and considered significant when *p* < 0.05 (*n* = 5–8 animals/group).

At P25, forebrain volumes were assessed using T_2_W images. HI and HILf10 had smaller volumes compared to Sham animals (F(4,36) = 6.661, *p* < 0.001) evidencing the tissue preservation using 0.1 and 1 g/kg doses (Figure 4a). The maps of brain diffusivities (Mean, MD; Axial, AD and Radial, AD), fractional anisotropy (FA) are presented in Figure 4b. NODDI-derived parameters, such as intra-neurite volume fraction (fin), cerebrospinal volume fraction (fiso), and orientation dispersion index (ODI) are presented in Figure S2. These parameters represent a good evaluation of microstructure integrity. High signal-to-noise ratio and very good resolutions were obtained leading to accurate measurement of the DTI and NODDI derived parameters, similar to those previously reported [42]. In the motor cortex, fiso was decreased in the posterior region of the motor cortex in the HI and HILf1 groups compared to Sham (H = 11.22, *p* = 0.02) (Figure S2). In the somatosensory cortex, no significant differences in the microstructure were observed (Figure S2). In the Cingulum, (Figure 4c—left lower panels) the HILf10 group showed increased AD (F(4,36) = 2.63, *p* = 0.05) and FA (F(4,36) = 2.80, *p* = 0.04) in the posterior region of the brain compared to the Sham group (F(3,17) = 3.44, *p* = 0.04). Moreover, in the posterior cingulum, FA was increased in the HILf10 group. The HI group presented an increase in RD and MD in the anterior region of the cingulum ((F(4,36) = 3.26, *p* = 0.02) and (F(4,36) = 2.63, *p* = 0.05), respectively). MD was also increased in the HILf10 group. Despite demonstrating no difference with Sham group, all HI treated groups displayed increased FA compared to the HI group in the anterior region of the cingulum (F(4,36) = 3.41, *p* = 0.02). No significant differences were observed in the internal capsule (Figure S2). In the caudate putamen, an increase in FA in groups HILf1 and HILf0.1 was observed ((F(4,36) = 3.25, *p* = 0.02) (Figure S2). One-way ANOVA performed in the posterior region of the corpus callosum (Figure 4d—central lower panels), showed an increase in AD in the HI and HILf10 g/kg groups ((F(4,36) = 3.74, *p* = 0.01). HILf0.1 had decreased FA in the anterior region of the CC compared to HILf1 and HILf10 (F(4,36) = 2.77, *p* = 0.04). In the anterior region of CC, AD, and MD were increased only in HILf10 animals (F(4,36) = 3.09, *p* = 0.02) and F(4,36) = 3.36, *p* = 0.01, respectively). In the external capsule, no differences were observed in the anterior region (Figure 4e—right lower panels). However, in the posterior region of the structure, MD and RD were increased in the HILf10 group ((F(4,36) = 2.84, *p* = 0.04) and (F(4,36) = 3.52, *p* = 0.01), respectively) and unchanged in HILf0.1 and HILf1 groups.

### 3.5. High Doses of Lf Increase Long-Term Astrogliosis in Myelinated Structures and Hypomyelination

**Figure 5:**
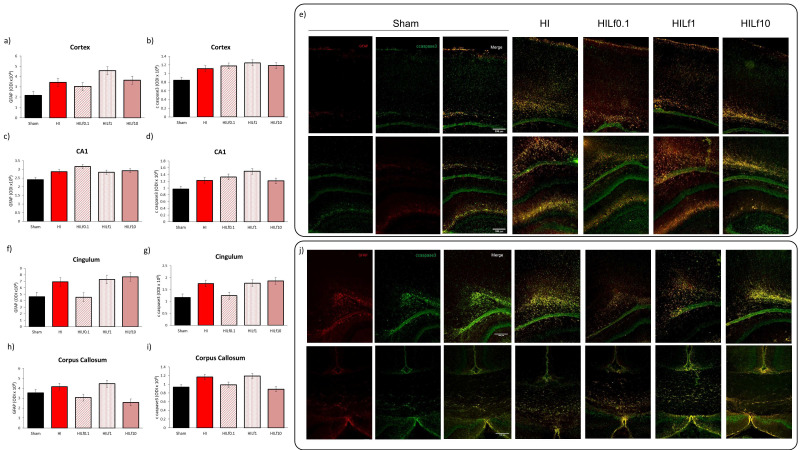
Optical density intensity (ODI) assessment of astrocytes (GFAP) and cleaved caspase 3 (apoptosis) in the cortex (**a** and **b**, respectively), CA1 (**c** and **d**, respectively), cingulum (**f** and **g**, respectively), and corpus callosum (**h** and **i**, respectively) in a 200 × 200 µm area in 30 µm brain slices at the dorsal hippocampus level at P4. Representative images of the brain regions assessed (**e**,**j**). Scale bar 100 µm. Differences were determined by one-way ANOVA and considered significant when *p* < 0.05 (*n* = 3–5 animals/group). No significant differences were observed.

**Figure 6:**
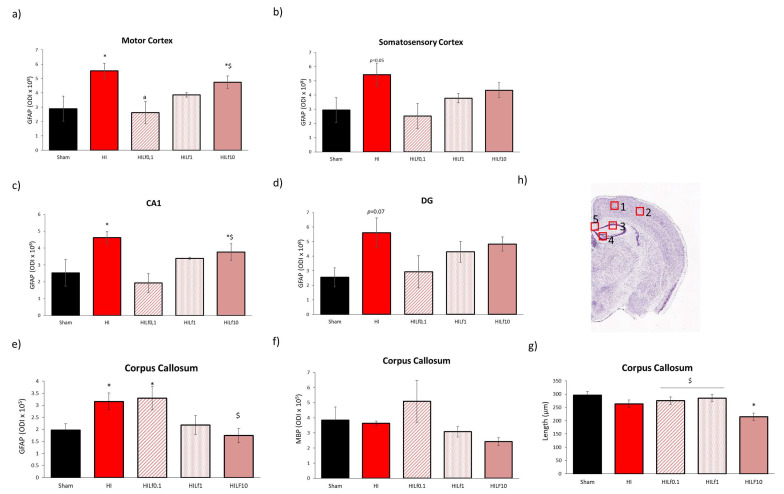
Effects of Lf and HI on brain tissue at P25. Optical density intensity (ODI) of astrocytes (GFAP) in the motor cortex (**a**), somatosensory cortex (**b**), CA1 (**c**), dentate gyrus (**d**), and corpus callosum (**e**). Assessment of myelination (MBP) and thickness (**f**,**g**) of the corpus callosum CC using 30 µm brain slices at the dorsal hippocampus level. Representative image of the brain regions assessed (**h**). Scale bar 100 µm. * HI vs. SH, ^$^ difference between HILf groups. ^a^ HILf0.1 vs. HI, respectively. Differences were determined by one-way ANOVA and considered significant when *p* < 0.05 (*n*= 3–5 animals/group).

At P4, despite the similar pattern observed in western blotting in the expression of GFAP and cleaved caspase 3 in the cortex (Figure 5a), cingulum (Figure 5e) and corpus callosum (Figure 5g), no statistical differences were observed in immunohistology evaluation including for the double labelling with NeuN and cleaved caspase 3 (Figure S3). Co-localization between GFAP and ccaspase3 in the CC evidenced an effect of injury (F(4,14) = 3.65, *p* = 0.04; HI animals had increased R values, indicating a higher co-localization between astrocytes and the apoptotic marker compared to the Sham group with no effects of Lf (data not shown). At P25, optical density intensity (ODI) of astrocytes (GFAP) in the right motor cortex, somatosensory cortex, CA1 and dentate gyrus (DG) were assessed (Figure 6a–d). One-way ANOVA showed an increase in reactive astrogliosis in HI animals reversed by Lf0.1 and 1 g/kg in the motor cortex (F(4,18) = 4.59, *p* = 0.01) and CA1 (F(4,18) = 4.45 *p* = 0.001) (Figure 6a and c, respectively). The same pattern was observed in the somatosensory cortex and DG, however, no statistical significance was seen (*p* = 0.05 and *p* = 0.07, respectively) (Figure 6b,d). Despite the same pattern of activation for GFAP, no differences were found in Iba-1 immunofluorescence in the regions analysed (data not shown). A decrease in NeuN immunostaining was observed, namely in the HILf10 group in the cortex and hippocampus, although not reaching statistical significance (data not shown). In the corpus callosum, HI and HILf0.1 animals had increased reactivity of GFAP compared to the other groups (F(4,15) = 3.98 *p* = 0.03) (Figure 6e). MBP assessment in the CC revealed no differences among groups (Figure 6f), however CC thickness was decreased in HI and HILf10 groups (F(4.15) = 3.93 *p* = 0.03), compared to Sham animals, pointing the neuroprotective effects of Lf administered at 0.1 and 1 g/kg of body weight through lactation.

## 4. Discussion

This study shows that neuroprotective effects of bovine lactoferrin through lactation at distinct doses (0.1, 1 and 10 g/kg body weight) causes early (24 h) and long-term (22 days) protection after neonatal HI insult in a dose-dependent manner. HIP3 caused a mild injury to the pup brain metabolism in the acute phase of injury. Lf was able to prevent energetic metabolic dysfunction at all dosages tested, although acting through different mechanisms. Our findings demonstrate that the protection afforded by Lf prevents primary energetic failure, preserving the coupling between astrocytes and neurons and the rise in inflammatory mediators, finally attenuating hypoxic–ischemic damage. Our data show that Lf reaches its optimal neuroprotection following HI at a dose of 1 g/kg.

After HI, an important marker of developmental damage is ponderal growth. Decreased body weight was observed in HI animals from P6 until end of experiment. Pups were weighed daily from P3 (before HI) to P6 and, despite no statistical difference, HI animals were smaller than diet-supplemented with Lf since beginning of experiments and all HI animals were smaller than controls. Body weight loss was not reversed with any dose of Lf. From P11, HI and HILf10 had smaller weights compared to the Sham group. Interestingly, at P25, HILf10 animals were smaller than Sham and HI rats, pointing to a delay or slackening growth rate, which could suggest a worse prognosis with the high Lf dose in the diet. In agreement, the literature shows that an increase in dietary proteins during pregnancy and lactation in mice induced the decrease in litter size as well as decreased the body weight of offspring [51]. In addition, after a 55% increase in maternal dietary protein, pups showed decreased food intake, impairment in glucose metabolism, and signs of insulin resistance [52].

MRS allows in vivo assessment of early alterations in the brain metabolism due to neonatal HI in preclinical and clinical setups with accuracy [53,54,55,56,57]. In the present study, we confirm previous data regarding HI and metabolite concentrations with a marked metabolic defect 24 h after injury evidencing the mild characteristic of the damage, in the absence of hyperintense signal on the T2W images [23]. HI caused a decrease in energetic metabolites (namely Cr, PCr), excitatory neurotransmission (Glu), and antioxidant defences (Asc), all prevented by Lf. The neurochemical profile analysis, especially regarding phosphocreatine (PCr) and glutamate (Glu), suggested that Lf modulates different metabolic routes in the early phase of injury depending on the dose administered. The lower dose of Lf (HILf0.1) preserved Glu/Gln (an index of neuron-astrocytic coupling functioning) that could be associated with better clearance of glutamate from the synaptic cleft, since levels of GSH were unaltered by Lf. This is also suggested by the increase in Gln, Lac, and Ins in the HILf10 group, which was not prevented by Lf. In agreement, Chen and colleagues showed that Lf modulates genes in a dose-dependent manner during early development of piglets in the absence of injury. Chen et al. showed that, when administered at low doses (i.e., 15 mg/kg/day), Lf modulated genes (associated) related to brain development, whereas higher doses (2 g/kg) should be used for neuroprotection [50]. Interestingly, our study shows that despite the molecular neuroprotection using the highest dose of Lf (10 g/kg), tissue damage was not reversed. This could be related to the dose, which is 5× higher than in Chen’s study.

Following HIP3, damage to the white matter is the hallmark of injury [23,58,59]. Strategies preventing hypomyelination have been shown to reduce brain injury and functional deficits; however, the mechanisms are still a matter of debate. In this context, astrocytes can have a major role since they contribute to energetic metabolism, maintaining ion homeostasis, cellular redox state [60,61,62] and glutamate uptake from the extracellular space converting it into glutamine or GSH [63]. GLT1 is the main CNS glutamate transporter [64] and is decreased in the HILf10 group during the early phase after HI whereas it remains unchanged in the two lower doses that could indicate astrocytic failure in glutamate reuptake, contributing to increased glutamatergic excitotoxicity observed at the high dose of Lactoferrin [65]. Allied to the decrease of IL-1β and GLT1 in the cortical tissue 24 h after injury, and to the late brain damage observed in HILf10, the data point to the interplay of oxidative stress, glutamatergic excitotoxicity, and neuroinflammation that is altered using the high Lf dose (for review, see [66]. Indeed, authors suggest that a decrease in inflammatory mediators early after injury are linked to increased oxidative injury, which could be the starting point of the glutamatergic excitotoxicity. Allied to this [67], lactoferrin decreases inflammation (TNF-α) in a dose-dependent manner, reaching an almost complete suppression of the release of the cytokine, when administered at 10 mg/kg. Previously, using the HIP3 model, Lf (1 g/kg) was able to decrease mRNA levels of TNF-α and IL-1β [40]. In the hippocampus (relatively spared in the HIP3 injury) [68] this pro-inflammatory profile was not observed, however, the increase in iNOS and synaptophysin could indicate a clear disbalance in excitatory transmission, leading to oxidative stress and necrotic cell death, worsening the initial injury (closely related to apoptotic cell death in HIP3 animals) [13]. The pathogenesis of brain injury is age dependent and particularly inflammatory processes appear to be important in the immature brain [69]. Increasing evidence indicates that TLR4 activation leads to oxidative stress after brain ischemia [70]. In our study, we observed an increase in TLR4 in the cortical tissue in HI treated groups, pointing to a possible role for Lf effects. In fact, TLR4 upregulation has been shown to initiate neuroinflammation and apoptosis via the NF-κB/IL-1β pathway after spinal cord injury [71], and Lf was able to suppress the TLR4 related pathway following cerebral ischemic reperfusion in mice [72].

Hypoxia–ischemia leads to increased production of oxidative species in the early phase after injury [73]. Lf is well known for its antioxidant and iron-chelating effects, reducing oxidative stress in the acute phase of HI injury [40]. In the current study, an increase in iNOS levels observed in the Lf treated groups could be explained by high affinity of Lf by free iron in the tissue, which could prevent oxidative reactions inducing acute cell death after HI. The evidence of early astrocyte dysfunction observed in our study points to astrocytic failure in solving oedema induced by HI. In this context, AQP4 is richly expressed in astrocytes and astrocytic swelling is an important early event in HI brain damage since AQP is responsible for regulating water movement into the cells [74]. AQP4 was decreased in HI and not different in the HI, HILf0.1, and HILf10 g/kg groups, and was increased in the HILf1 g/kg in the cortex 24 h after injury and highly increased in the hippocampus of the HILf0.1 group. AQP4 depletion has been found to protect adult mice brains from oedema [75,76], which may be part of a self-protective response to HI, to reduce further water accumulation in astrocytes and counteract the evolution of oedema. On the other hand, AQP4 overexpression has also been found to decrease cytotoxic oedema in HI models [77]. In agreement, the resolution of brain oedema and in the clearance of brain water from the tissue in the peri-infarct area in models of focal ischemia [78].

Long-term analysis of brain microstructure was performed using ex vivo advanced diffusion imaging at P25. Forebrain volumes were decreased in HI and HILf10 groups confirming the pattern of injury observed at P4 in the ^1^H-MRS and western blotting analysis. Injury at P3 leads to subsequent abnormal development of the brain [22]. Nevertheless, in our study, microstructural analysis of cortical areas did not reveal major changes in somatosensory and motor regions, confirming the mild nature of HIP3 [59,79], and this result matched well with the absence of T_2_W hypersignal 24 h post-injury. Indeed, in this model it has been shown [57] that the loss of cortical structure at P25 was correlated with the size (level) of the lesion at P4. Furthermore, DTI-NODDI derived parameters were mildly altered in myelinated structures, such as cingulate gyrus, external capsule and corpus callosum. In these white matter regions, diffusivity values were increased or tended to increase confirming the altered microstructure, however this effect was reversed mainly for the HILf1 group. The pattern observed in white matter of HI animals showed increased axial and radial diffusivities combined with reduced fin, suggesting higher mobility of water with less barriers, which could be related to reduced axonal density, also shown by decreased MBP staining. In agreement with the histological analysis presented, previous studies observed changes in parallel diffusivity and fractional anisotropy on diffusion tensor imaging in the corpus callosum, and internal capsule in newborn rabbits following intrauterine inflammation, which could be explained by the changes in astrocyte and microglial morphology seen in the white matter tracts, which is a hallmark of preterm HI damage [80]. The decrease observed in parallel diffusivity and fractional anisotropy MRI analysis of forebrain showed that HILf10 g/kg had cerebral damage and altered development similar to HI animals. This effect was confirmed by the histological analysis, in which the HILf10 group presented decreased corpus callosum length and MBP expression evidencing white matter damage. Interestingly, patients suffering from gelatinous drop corneal dystrophy (an autosomal recessive genetic disease that can occur at an early age and cause growth delays) exhibit high levels of Lf gene expression in their corneal tissue, which can correlate excess Lf to developmental delays and might help explain the detrimental effects of Lf [81,82].

## 5. Conclusions

The neuroprotective effects of Lactoferrin have been attracting widespread attention among clinical and preclinical researchers in the last years [50,83]. Kaufman and colleagues administered up to 300 mg/day of enteral bovine Lf and reported no adverse effects in preterm babies [83]. Although in general they related to pathways involving inflammation, the molecular mechanisms underlying the physiological changes induced by different concentrations of Lf administered and its effects on the CNS are very scarce. In this study, bovine lactoferrin had the ability of modulating distinct molecular signalling pathways in the CNS after an HI insult to the early developing brain, according to the Lf dose received during lactation. Despite early neurochemical protection, high levels of Lf in the diet can induce deleterious effects through alteration of brain response to HI, which reinforces the importance of this study. Furthermore, it is important to notice that despite evidence of neuroprotection shown here, behavioural assessment was not in the scope of the study and certainly needs further investigation. Neonatal HI is a complex pathology in which the primary damage can result in functional outcomes, ranging from almost no detectable injury to severe neurological deficits. Our data suggest that neuroprotection induced by Lf in the primary phase of injury, reducing apoptosis, inflammation, oxidative stress, and glutamatergic excitotoxicity, can be mitigated by the administration of an adequate dose of lactoferrin.

## Figures and Tables

**Table 1:** List of primary antibodies used. List of abbreviations for the antibodies: AQP4, aquaporin 4; ccaspase 3, cleaved caspase 3; DCX, doublecortin; GFAP, glial fibrillar acid protein; GLT1, glutamate transporter 1; Iba-1, ionised calcium binding adaptor molecule 1; IL-1 β, interleukin 1 beta; iNOS, inducible nitric oxide synthase; TLR4, toll-like receptor 4.

Antibody	Company	Reference	Host	Molecular Weight
Actin	Millipore	MAB1501	Mouse	42 kDa
AQP4	Abcam	ab125049	Rabbit	35 KDa
ccaspase 3	Cell Signalling	9661	Rabbit	19 kDa
ccaspase 3	Abcam	ab214430	Rabbit	17 KDa
DCX	Abcam	ab18723	Rabbit	45 kDa
Fractin	Millipore	aB3150	Rabbit	32 kDa
GFAP	Sigma	G6171	Mouse	50 KDa
GLT1	Abcam	ab106289	Rabbit	62 kDa
Iba-1	Abcam	ab5076	Goat	17 KDa
IL-1β	Abcam	ab254360	Rabbit	30 KDa
iNOS	Abcam	Ab15323	Rabbit	140 KDa
NeuN	Millipore	MAB377	Mouse	46/48 kDa
Synaptophysin	Abcam	Ab8049	Mouse	34 KDa
TLR4	Abcam	Ab22048	Mouse	90 KDa
Tubulin	Abcam	Ab18207	Rabbit	50/55 kDa

**Table 2:** Pup body weight monitoring during the experimental period. Litters were composed of 10 animals during experiments. Average weight (grams). ^†^ One-way repeated measures ANOVA * HI vs. Sham; ^$^ effect of lactoferrin dose—HILf10 vs. HILf1; ^a c^ HILF0.1 or 10 vs. HI, respectively. Intra-day differences were determined by one-way ANOVA and considered significant when *p* < 0.05.

	P3	P4	P5	P6 ^†^	P11 ^†^	P18 ^†^	P25 ^†^
Sham (*n* = 10)	9.31 ± 1.15	10.95 ± 1.07	13.19 ± 1.51	15.24 ± 1.43	25.8 ± 2.15	41.4 ± 2.91	74.12 ± 6.08
HI (*n* = 18)	8.88 ± 1.10	10.01 ± 1.44	11.88 ± 1.86	13.48 ± 2.22 ^.06^	23.2 ± 2.62 *	38.06 ± 4.23	67.81 ± 8.97 *
HILf0.1 (*n* = 29)	9.25 ± 1.56	10.62 ± 1.43	12.00 ± 1.77	13.93 ± 1.73	23.79 ± 2.94	36.69 ± 4.55 *^a^	65.05 ± 8.20 *^a^
HILf1 (*n* = 20)	9.44 ± 0.50	10.41 ± 0.78	11.90 ± 1.17	13.75 ± 1.55	23.65 ± 2.13	38.65 ± 2.92	67.27 ± 6.86 *
HILf10 (*n* = 27)	9.17 ± 0.87	10.11 ± 1.02	11.67 ± 1.33	13.11 ± 1.36 *	22.52 ± 1.78 *	36.56 ± 3.22 *^c^	66.42 ± 6.79 *^c$^

## Data Availability

All data here presented may be made available upon request to the authors.

## Supplementary Materials

The following are available online at https://www.mdpi.com/article/10.3390/nu13113880/s1, Figure S1: Effects of Lf and HI on protein expression in the right hippocampus at P4, (24 h after injury). Dams were fed control diet (Sham and HI), Lf 0.1 (HILf0.1), 1 (HILf1) or 10 (HILf10) g/kg lactoferrin. All proteins were quantified by densitometry, and then normalized to actin or tubulin and expressed as a value (ODI) relative to the Sham group for: fractin (a), cleaved caspase 3 (b), IL-1β (c), TLR4 (d), GFAP (e), AQP4 (f), GLT1 (g), iNOS (h), synaptophysin (i) and DCX (j). Representative bands of the proteins with the respective molecular weight (mw) in KDa (k). All values are presented as the mean ± SEM, *n* = 4–6 animals per group. * HI vs. SH, $ difference between HILf groups. a b c HILf0.1, 1 or 10 vs. HI, respectively. Differences were determined by one-way ANOVA and considered significant when *p* < 0.05. Figure S2: Optical density intensity (ODI) analysis of neurons (NeuN) and cleaved caspase 3 (apoptotic marker) in the cortex (a,b) and CA1 (c,d) at P4. Optical density intensity (ODI) analysis of astrocytes (GFAP) and Fractin (apoptotic marker) in the CC (e,f) at P4. Assessements were performed in a 200 × 200 µm area using 30 µm brain slices at the dorsal hippocampus level. Representative images of the brain regions assessed (g). Scale bar 100 µm. ODI values were compared by one-way ANOVA (*n* = 3 animals/group). No statistical differences were observed. Figure S3: MRI derived data at P25. NODDI-derived parameters in the cingulate gyrus (left upper panels), corpus callosum (central upper panels) and external capsule (right upper panels). DTI/NODDI in the internal capsule (left lower panels), motor cortex (central lower panels) and somatosensory cortex (right lower panels). Diffusivity (Mean, MD; Axial, AD; Radial, RD), fractional anisotropy (FA) and direction encoded color (DEC) maps, intra-neurite volume fraction (fin), intra-restricted volume fraction (irfrac), isotropic volume fraction (fiso), and orientation dispersion index (ODI) maps at P25 for each group. Lower panels: Histogram of diffusivities (Mean, MD; Axial, AD and Radial, RD; ×10−4 mm^2^·s−1), fractional anisotropy (FA), intra-neurite volume fraction (fin), cerebrospinal volume fraction (fiso), and orientation dispersion index (ODI). Results are mean ± SEM. * HI vs. SH, $ difference between HILf groups. a b c HILF0.1 1 or 10 vs. HI respectively as determined by one-way ANOVA. (*n* = 5–8 animals/ group).
