# Supplementary material for: Dose-Dependent Neuroprotective Effects of Bovine Lactoferrin Following Neonatal Hypoxia–Ischemia in the Immature Rat Brain
Source: Nutrients. 2021 Oct 29;13(11):3880. doi: 10.3390/nu13113880 (PMC8618330; doi:10.3390/nu13113880)
Supplement: Supplementary file 1 [file nutrients-13-03880-s001.zip › nutrients-1415802-supplementary.pdf]

Figure S1

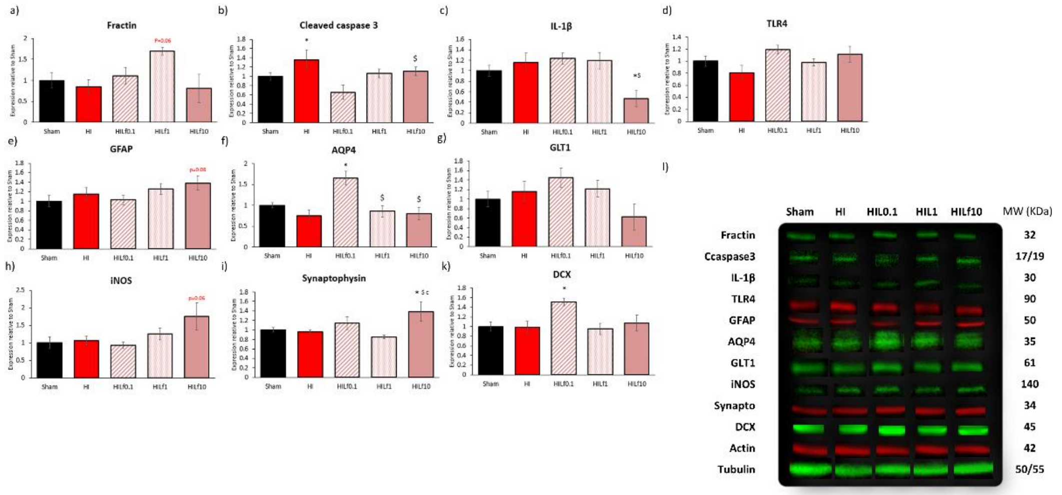

Figure S1 - Effects of Lf and HI on protein expression in the right hippocampus at P4, (24h after injury). Dams were fed control diet (Sham and HI), Lf 0.1 (HILf0.1), 1 (HILf1) or 10 (HILf10) g/kg lactoferrin. All proteins were quantified by densitometry, and then normalized to actin or tubulin and expressed as a value (ODI) relative to the Sham group for: fractin (a), cleaved caspase 3 (b), IL-1β (c), TLR4 (d), GFAP (e), AQP4 (f), GLT1 (g), iNOS (h), synaptophysin (i) and DCX (j). Representative bands of the proteins with the respective molecular weight (mw) in KDa (k). All values are presented as the mean±SEM, *n*=4-6 animals per group. * HI vs. SH, $ difference between HILf groups. a b c HILf0.1, 1 or 10 vs HI respectively. Differences were determined by one-way ANOVA and considered significant when $p<0.05$.

Figure S2

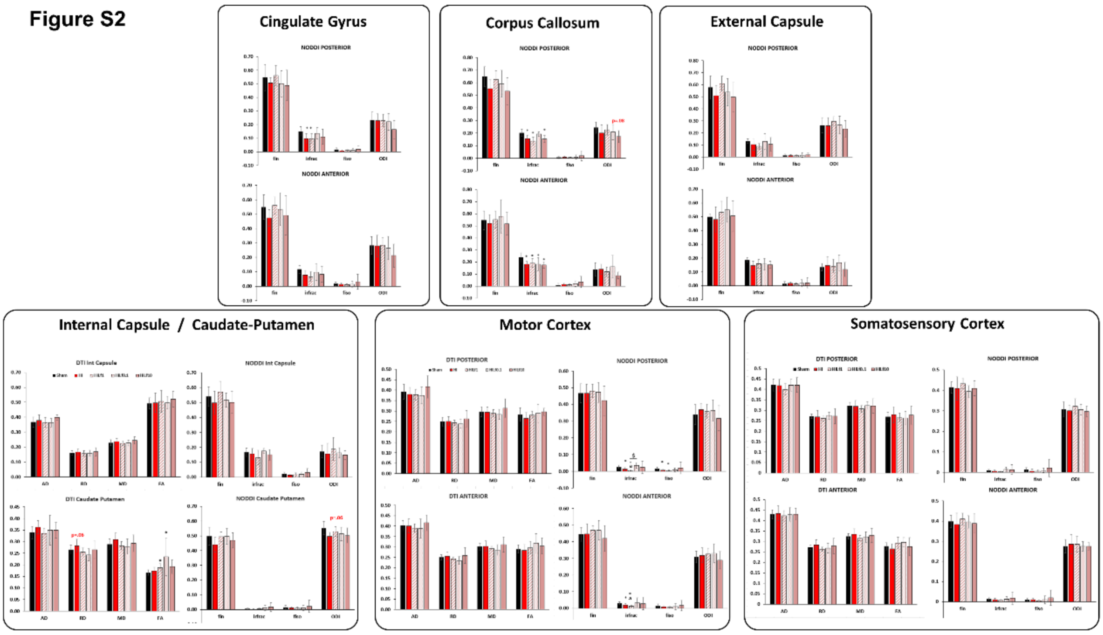

Figure S2 - Optical density intensity (ODI) analysis of neurons (NeuN) and cleaved caspase 3 (apoptotic marker) in the cortex (a-b) and CA1 (c-d) at P4. Optical density intensity (ODI) analysis of astrocytes (GFAP) and Fractin (apoptotic marker) in the CC (e-f) at P4. Assessements were performed in a 200x200 µm area using 30µm brain slices at the dorsal hippocampus level. Representative images of the brain regions assessed (g). Scale bar 100 µm. ODI values were compared by one-way ANOVA (n= 3 animals/ group). No statistical differences were observed.

Figure S3

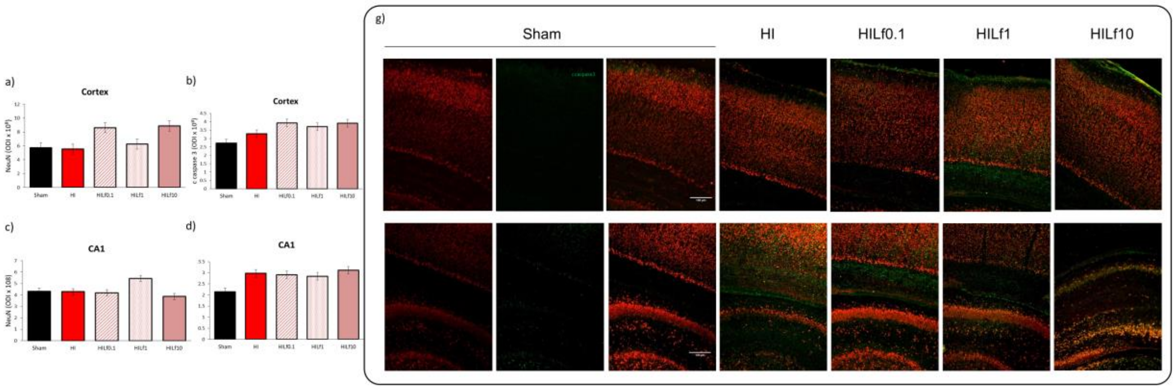

Figure S3 - MRI derived data at P25. NODDI-derived parameters in the cingulate gyrus (left upper panels), corpus callosum (central upper panels) and external capsule (right upper panels). DTI/NODDI in the internal capsule (left lower panels), motor cortex (central lower panels) and somatosensory cortex (right lower panels). Diffusivity (Mean, MD; Axial, AD; Radial, RD), fractional anisotropy (FA) and direction encoded color (DEC) maps, intra-neurite volume fraction (fin), intra-restricted volume fraction (irfrac), isotropic volume fraction (fiso), and orientation dispersion index (ODI) maps at P25 for each group. Lower panels: Histogram of diffusivities (Mean, MD; Axial, AD and Radial, RD; $\times 10^{-4}$ mm2.s−1), fractional anisotropy (FA), intra-neurite volume fraction (fin), cerebrospinal volume fraction (fiso), and orientation dispersion index (ODI). Results are mean ± SEM. * HI vs. SH, $^{\$}$ difference between HILf groups. $^{a\ b\ c}$ HILF0,1 1 or 10 vs HI respectively as determined by one-way ANOVA. (n- 5-8 animals/ group).
